# Safety of Venous Thromboembolism Prophylaxis Protocol Using a Novel Leg Exercise Apparatus in Bedridden Patients due to Spinal Diseases

**DOI:** 10.7759/cureus.19136

**Published:** 2021-10-29

**Authors:** Kanami Kobayashi, Yukiyo Shimizu, Ayumu Haginoya, Yasushi Hada, Masashi Yamazaki

**Affiliations:** 1 Clinical Science, Graduate School of Comprehensive Human Science, Tsukuba, JPN; 2 Rehabilitation Medicine, University of Tsukuba, Tsukuba, JPN; 3 Orthopaedic Surgery, University of Tsukuba, Tsukuba, JPN

**Keywords:** spinal disease, bed rest, symptomatic venous thromboembolism, venous thromboembolism prophylaxis, leg exercise apparatus

## Abstract

Purpose: Venous thromboembolism (VTE) is a complication in patients with a spinal disease requiring bedfast for conservative therapies. We previously developed a novel leg exercise apparatus (LEX) to encourage patients to exercise their lower extremities during bed rest. The purpose of this study was to evaluate the feasibility and safety of the LEX for the prevention of VTE in patients on bed rest due to spinal disease.

Methods: Patients with spinal diseases requiring bed rest were included in the study. Exercise using the LEX was performed for ≥5 minutes. The exercises were performed three or more times per day during the bed rest period. In addition, we evaluated adverse events, such as symptomatic VTE and changes in vital signs, using venous ultrasonography, blood tests, and measurement of vital signs.

Results: In total, 31 patients were enrolled (11 men, 20 women), with mean age, height, weight, and body mass index of 72.4 years, 155.2 cm, 55.0 kg, and 22.6 kg/m^2^, respectively. Twenty-four subjects had spinal fractures. Twenty-nine patients continued exercising until they could leave their beds. No symptomatic VTE was observed in any patient, and no other severe adverse events were observed. There were no significant changes in vital signs. The average number of exercise days with LEX and length of hospitalization were 11 and 31 days, respectively.

Conclusions: This is the first study regarding mechanical thromboprophylaxis through in-bed exercise for patients with bedridden spinal disease. The LEX exercise protocol, in addition to mechanical prophylaxis with graduated compression stockings and intermittent pneumatic compression devices, for the prevention of symptomatic VTE may be feasible and safe for patients with bedridden spinal disease.

## Introduction

Venous thromboembolism (VTE) is a crucial problem for bedridden patients. Ota et al. studied the incidence of VTE and reported its remarkable increase in Japan from 1996 to 2011 [1]. In particular, orthopedic patients are at a higher risk of VTE among all patients, and those who undergo spinal surgery are at risk of VTE [2-5]. Moreover, patients who underwent spine fracture surgery have a higher VTE rate of 9.2% than that of other spine patients' VTE rate of 2.3%. [6]. A longer hospital stay and older age are associated with greater risks of VTE events among patients with spinal fractures [7].

Mechanical and pharmacological thromboprophylaxis is recommended in the guidelines of the American College of Chest Physicians (ACCP) [8]. However, pharmacological prophylaxis does not apply to patients with a risk of bleeding [8]. Mechanical prophylaxis is recommended rather than pharmacological methods for patients undergoing spinal surgery to prevent bleeding complications. Mechanical prophylaxis includes graduated compression stockings (GCS), intermittent pneumatic compression device (IPCD), early ambulation, and voluntary lower extremity exercises. Mechanical thromboprophylaxis focuses on the stasis of blood flow, which is one of Virchow’s triad [9,10]. The major disadvantage of GCS and IPCD is poor patient compliance [9]. Patients may have difficulties continuing to use these devices due to side effects such as lower extremity pain and skin problems, including pressure ulcers and itching [9]. It is difficult to get out of bed early in patients with spinal fractures or before spinal surgery because they are forced to be bedfast for treatment. Although we recommend voluntary lower extremity exercises to prevent VTE, patients with spinal disease rarely perform leg exercises voluntarily. Therefore, we developed a novel leg exercise apparatus (LEX) that helps patients move their lower extremities voluntarily [11-14]. We found that a brief period of exercise using the LEX increased venous flow in the lower extremities compared with the continuous use of IPCD [12].

This study aimed to evaluate the feasibility and safety of using a novel LEX for the prevention of VTE in bedridden patients with spinal diseases.

## Materials and methods

Ethical consideration

This study was carried out as per the principles of the Declaration of Helsinki and was approved by the institutional review board of the hospital (No. H29-182). All participants provided verbal and written informed consent before completing any study-related tasks.

Participants

Patients with a spinal disease requiring bed rest were enrolled between January 2019 and May 2021. The exclusion criteria were as follows: 1) patients with a history of proximal deep vein thrombosis (DVT); 2) patients with spinal instability even in bedfast positions; 3) patients with a cerebral aneurysm or dissecting aortic aneurysm, wherein an increase in blood pressure may aggravate their general condition; 4) patients with severe complications (e.g., heart disease, liver disease, and kidney disease); 5) patients who were pregnant or suspected of being pregnant; 6) patients who were unable to consent voluntarily; and 7) others who were considered ineligible by the attending physician.

Leg exercise apparatus (LEX)

**Figure 1:**
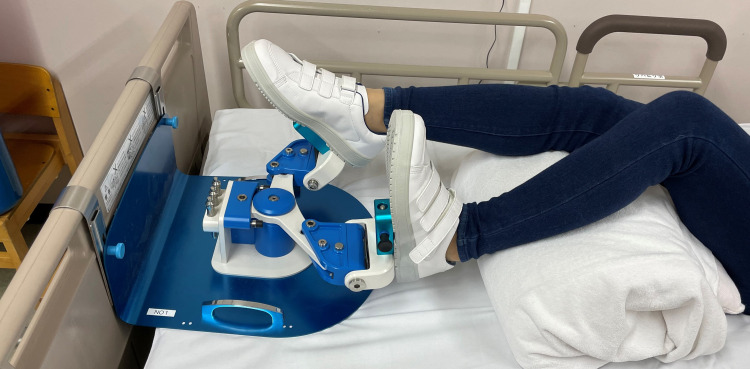
The LEX The LEX is made up of right and left pedals, shoes, a metal base, hooks, and a motion control mechanism. LEX: Leg exercise apparatus

The LEX is a device developed to facilitate active lower extremity exercise and consequently prevent VTE. Patients can perform leg exercises effectively using the LEX as it was designed to perform leg exercises in the supine position (Figure 1) [11-14]. The device is made up of right and left pedals and a motion control mechanism, which can be placed on the bed and fixed to the backboard of the bed by hooks. The feet are fixed to the pedal using shoes. The patient’s ankles can move up to 30° dorsiflexion, 60° plantar flexion, 30° subtalar inversion, and 20° subtalar eversion when the feet are fixed on the pedal. The LEX does not inhibit the performance of the ankle, knee, and hip joints. We tried to maintain the patients’ knee flexion position during exercises with the LEX to make contraction of the soleus muscle dominant over that of the gastrocnemius muscle.

Primary outcomes

To investigate the feasibility and safety of performing exercises with the LEX, we evaluated the presence or absence of adverse events related to exercise using the LEX. We assessed symptomatic VTE and other adverse events, such as death, death from complications, and prolonged hospital stay, to treat complications and disability due to complications.

The presence of DVT was assessed by ultrasonography of the lower extremities and blood tests, including D-dimer levels, both before starting the LEX protocol and after the LEX protocol.

Secondary outcome

To investigate the feasibility of exercises with the LEX, we measured the effect on circulatory dynamics, including blood pressure, pulse rate, and percentage saturation of oxygen before and after exercise on the first and last days of the exercise. In addition, the maximum calf circumference and the thigh circumference 10 cm above the patella were measured on the first and last days of exercise. Moreover, the degree of fatigue was evaluated using the modified Borg scale, before and after LEX exercise in both first and last sessions (Table 1) [15].

**Table 1:** The modified Borg scale - a subjective estimate of the degree of fatigue

Modified Borg scale	
0	No breathlessness
0.5	Very, very slight (just noticeable)
1	Very slight
2	Slight breathlessness
3	Moderate
4	Somewhat severe
5	Severe breathlessness
6	
7	Very severe breathlessness
8	
9	Very, very severe (almost maximum)
10	Maximum

Statistical analyses

Paired t-tests were performed to compare D-dimer levels before and after the LEX intervention, changes in vital sign changes (systolic and diastolic blood pressure, pulse rate, and percentage saturation of oxygen) before and after LEX use on the first and last days of exercise, and circumferences of both the calf and thigh on the first and last days of the LEX intervention. Modified Borg Scales before and after LEX use on the first and last days were tested using the Wilcoxon signed-rank test. Statistical significance was set at p<0.05. Analyses were performed using JMP 14.3.0 (SAS Institute Inc., Cary, NC, USA).

Intervention

Participants performed active leg exercises using the LEX in the supine position to avoid load on the spine and spine torsion and to maintain the stability of the spinal column. We evaluated whether each participant could easily move their legs while in the supine position and would, if necessary, place a cushion under their knees to increase the ease at which they could move their legs. We did not set the pace for exercises using the LEX. Instead, participants determined the pace at which they could comfortably move their legs. The exercise sessions lasted for at least five minutes and were repeated three times a day. Shimizu et al. showed that one minute of exercise with the LEX had a lasting effect of 30 minutes and stated that she would conduct a future exercise with the LEX four times a day for five minutes each time [15]. In this study, we decided that these exercise sessions should be five minutes in duration. However, the number of sessions was limited to three per day, as this was considered feasible in our clinical setting. Physical therapists and nurses or assistant nurses supervised the sessions once a day and twice per day on weekdays, respectively. The patients performed the exercises under the supervision of nurses or assistant nurses three times a day on weekends. The exercise program started after confirming there was no symptomatic VTE and finished when patients could get out of bed or thereafter. A physician or physical therapist supervised the first exercise session and guided how to perform the exercises correctly to avoid improper movements. A physician or physical therapist also decided how to set the device and cushion and explained the optimal position of the participant to the nurses.

Management and assessment to prevent VTE during the bed rest period

Assessments and exercises were conducted following the procedure shown in Table 2.

**Table 2:** Interventional procedures LEX: Leg exercise apparatus

Day	Procedure	
Pre LEX	Informed consent	
	D-dimer	
	Ultrasonography	
	Practice exercise using LEX	
The first day of LEX (before LEX)	Blood pressure, pulse rate, and percentage saturation of oxygen	
	Modified Borg scale	
The first day of LEX (after LEX)	Blood pressure, pulse rate, and percentage saturation of oxygen	
	Modified Borg scale	
LEX period	Exercise using LEX three times per day	
Before the last day of LEX	D-dimer	
	Ultrasonography	
The last day of LEX (before LEX)	Blood pressure, pulse rate, and percentage saturation of oxygen	
	Modified Borg scale	
The last day of LEX (after LEX)	Blood pressure, pulse rate, and percentage saturation of oxygen	
	Modified Borg scale	

Graduated compression stockings were used in all cases to prevent VTE, while IPCD was also used in cases considered without DVT. Due to atrial fibrillation, edoxaban was used in one case, and rivaroxaban was used in another. The participants used GCS for the entire bed rest period. The IPCD was detached during exercise with LEX.

Cancellation criteria

Patients were excluded from the study if any of the following events occurred: 1) adverse events that make it difficult to continue research; 2) requests from the patient to withdraw from participation in the study; 3) withdrawal of consent for participation in the study; 4) discovery of continuous violations to the study plan; 5) judgment on discontinuation by the attending physician for some reason.

## Results

In total, 31 patients were enrolled, including 11 men and 20 women, with a mean age, height, weight, and body mass index (BMI) of 72.4 years (range: 22 to 91 years), 155.2 cm (range: 142 to 171 cm), 55.0 kg (range: 35.0 to 100.7 kg), and 22.6 kg/m2 (range: 15.4 to 34.8 kg/m2), respectively (Table 3). None of the patients met any of the exclusion criteria, and all consented to participate in the study. Twenty-four patients had spinal fractures. Overall, 29 of the 31 patients completed the exercise regimen. No serious adverse events occurred during the study period. The average number of LEX exercise days was 11 (range: three to 33 days), and the average length of hospitalization was 31 days (range: 13 to 64 days).

**Table 3:** Background of the patients enrolled in this study BMI: Body mass index

Subject	Sex	Age	Height [cm]	Weight [kg]	BMI [kg/m^2^]	Diagnosis	Surgery
1	Female	63	143.0	44.9	22.0	Postoperative infection (Th9,10)	+
2	Male	76	165.0	76.0	27.9	Vertebral fracture (Th9)	+
3	Male	76	168.0	52.8	18.7	Vertebral fracture (L1)	+
4	Male	80	171.0	67.0	22.9	Myelopathy (C4,5)	+
5	Female	64	151.5	56.0	24.4	Vertebral fracture (Th6)	+
6	Female	77	144.0	49.6	23.9	Vertebral fracture (Th12)	+
7	Female	63	151.0	44.2	19.4	Vertebral fracture (Th12)	-
8	Male	86	165.0	64.5	23.7	Vertebral fracture (Th4)	-
9	Female	75	152.0	63.5	27.5	Vertebral fracture (L1)	-
10	Female	83	151.2	36.6	16.0	Vertebral fracture (Th12)	-
11	Female	85	142.0	42.4	21.0	Vertebral fracture (L1)	-
12	Female	91	144.0	42.1	20.3	Vertebral fracture (L1)	-
13	Male	84	160.0	45.3	17.7	Vertebral fracture (L1)	+
14	Female	70	154.0	52.0	21.9	Vertebral fracture (L1)	-
15	Male	65	165.0	77.2	28.4	Vertebral fracture (L4)	-
16	Male	67	164.4	63.4	23.5	Vertebral metastasis (Th4.5.6)	+
17	Female	78	151.0	35.0	15.4	Pseudoarthrosis (L2)	+
18	Female	67	153.0	62.0	26.5	Vertebral fracture (Th10)	+
19	Female	72	148.0	38.4	17.5	Vertebral osteomyelitis (L4.5)	+
20	Female	79	151.2	49.1	21.5	Vertebral fracture (L1)	-
21	Female	69	149.0	51.9	23.4	Vertebral fracture (Th9)	+
22	Male	80	152.5	57.1	24.6	Vertebral fracture (L1)	-
23	Male	71	158.8	55.1	21.8	Vertebral fracture (Th12)	+
24	Male	60	166.0	67.8	24.6	Vertebral fracture (Th12)	-
25	Female	71	154.0	52.0	21.9	Vertebral fracture (Th11, L1)	-
26	Female	76	156.0	39.2	16.1	Vertebral fracture (L1)	-
27	Female	72	155.0	45.7	19.0	Vertebral osteomyelitis (L4.5)	-
28	Female	82	149.3	53.4	24.0	Vertebral fracture (Th7)	-
29	Male	59	170.2	100.7	34.8	Ossification of ligamentum flavum (Th10, 11)	+
30	Female	22	160.0	59.0	23.0	Vertebral fracture (L1,3,4)	-
31	Female	81	147.0	60.2	27.9	Vertebral fracture (S)	-

**Figure 2:**
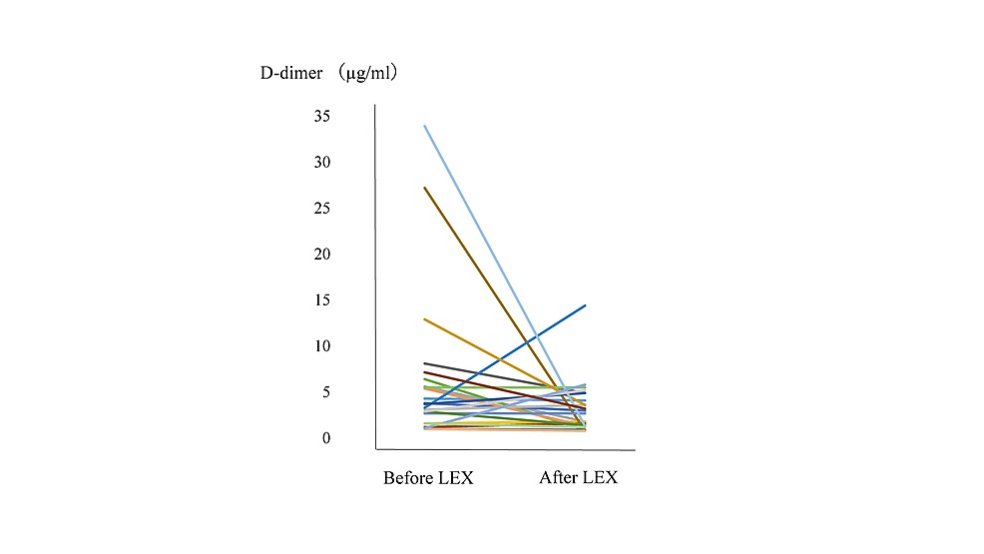
Changes in D-dimer level before starting the LEX protocol and after the LEX protocol LEX: Leg exercise apparatus

No symptomatic VTEs were observed following the LEX protocol. Twenty of the 31 patients underwent ultrasonography of the lower extremities before getting out of the bed due to the risk of blood clots, and no new DVTs were observed. The mean D-dimer level before the LEX program was 7.1 μg/mL, whereas the corresponding value following the LEX program was 3.2 μg/mL (Figure 2). Paired t-test analysis did not show a significant difference in D-dimer levels before or after LEX treatment (p=0.173).

The average systolic blood pressure, diastolic blood pressure, pulse rate, and SpO2 changed from 130.4 to 130.9 mmHg, 78.0 to 79.9 mmHg, 78.8 to 78.9 bpm, and 96.9 to 96.8 %, respectively, before and after the first exercise session. The average systolic blood pressure, diastolic blood pressure, and pulse rate changed from 125.5 to 127.1 mmHg, 69.9 to 70.4 mmHg, and 74.7 to 76.7 bpm, respectively, while the average SpO2 did not change, before and after the last exercise session. Table 4 shows the mean circulatory dynamics, including blood pressure, pulse rate, and percentage saturation of oxygen. Paired t-test analysis showed a significant difference in diastolic blood pressure before and after the first exercise session (p=0.047).

**Table 4:** Comparison of blood pressure, pulse rate, and the percentage saturation of oxygen before and after exercise using the LEX in the first and last exercise sessions Data are presented as mean. Paired t-tests were performed. *p <0.05. 'Before' column features data collected before the LEX exercise. 'After' column features data collected after the LEX exercise. sBP: Systolic blood pressure; dBP: Diastolic blood pressure; PR: Pulse rate; SpO2: Saturation of percutaneous oxygen; LEX: Leg exercise apparatus

BP, PR, and SpO2 in the first exercise session				
	Before	After	p-value	N
sBP [mmHg]	130.4	131.6	0.446	28
dBP [mmHg]	78.0	80.5	0.047*	27
PR [bpm]	78.8	79.4	0.445	28
SpO2 [%]	96.9	96.9	1.000	26
BP, PR, and SpO2 in the last exercise session				
	Before	After	p-value	N
sBP [mmHg]	125.5	127.1	0.152	18
dBP [mmHg]	69.9	70.4	0.667	18
PR [bpm]	74.7	76.7	0.132	18
SpO2 [%]	97.8	97.8	1.000	18

Circumference of the lower limbs in the first and last exercise sessions was assessed (Table 5.). The average maximum right and left calf circumferences changed from 30.1 to 30.7 cm and 30.2 to 30.8 cm, respectively, between the first and last exercise sessions. The average right and left thigh circumference 10 cm above the patella changed from 37.6 to 38.5 cm and 37.6 to 38.2 cm, respectively. Paired t-test analysis did not show a significant difference in the circumference of the lower limbs between the first and last exercise sessions using LEX.

**Table 5:** Comparison of the maximum calf circumference and the thigh circumference 10 cm above the patella on the first day and the last day of the LEX exercise Data are presented as mean. Paired t-test was performed; p*<0.05. 'First day' column features data collected on the first day of the LEX exercise. 'Last day' column features data collected on the last day of the LEX exercise. The maximum circumference of the calf was taken. The thigh circumference was taken 10 cm above the patella.

	First day	Last day	p-value	N
Right calf [cm]	30.9	30.7	0.780	22
Left calf [cm]	31.0	30.8	0.672	22
Right thigh [cm]	38.7	38.5	0.277	22
Left thigh [cm]	38.7	38.2	0.071	22

During the first exercise session, the average modified Borg scale scores for breaths and legs changed from 0.73 to 1.45 and 1.13 to 2.43, respectively. During the last exercise session, the average modified Borg scale scores for breath and legs changed from 0.55 to 0.71 and 0.55 to 0.95, respectively. Table 6 shows the median data regarding the modified Borg scale and p-values obtained using the Wilcoxon signed-rank test. The Wilcoxon signed-rank test showed a significant difference in the modified Borg scale scores for breaths and legs before and after exercise in the first exercise session (p=0.001 and p<0.001, respectively). A significant difference was also observed in the modified Borg scale scores for legs before and after exercise in the last exercise session (p=0.015).

**Table 6:** Comparison of the modified Borg scale before and after exercise using LEX in the first and last exercise sessions Data are presented as median. The Wilcoxon signed-rank test was performed; p*<0.05. 'Before' column features data collected before the LEX exercise. 'After' column features data collected after the LEX exercise.

Modified Borg scale in the first exercise session				
	Before	After	p-value	N
Breath	0	0.75	0.001*	28
Leg	0.25	2	<0.001*	28
Modified Borg scale in the last exercise session				
	Before	After	p-value	N
Breath	0	0	0.090	21
Leg	0	1	0.015*	21

Details of D-dimer levels, blood pressure, pulse rate, percentage oxygen saturation, lower extremity circumference, and modified Borg scale scores are shown in Appendix A.

## Discussion

In this study, all patients were able to perform the LEX exercise program without severe adverse events, including symptomatic VTE. Twenty-nine of the 31 enrolled patients completed the program. One patient withdrew due to back pain. She could not follow the rest of the instructions and, as such, walked around her room during the bed rest period. Another patient could not complete the program due to exacerbation of chronic renal failure. Although it is important to continue active lower extremity exercises to prevent thrombosis in patients with chronic renal failure, it was difficult for this patient to continue the exercises with the LEX due to the worsening of her general condition and the introduction of dialysis treatment. She only exercised using the LEX twice due to low motivation before the worsening of her chronic renal failure. Therefore, we did not think that the LEX exercise had a negative effect on her general condition. Active lower extremity exercise with LEX is considered painless for patients whose general condition is calm and who can observe the resting level restriction. Due to the limited pain during LEX exercise, it might be feasible to introduce this method to patients who are required to stay in bed.

Twenty-four of the 31 patients had vertebral fractures, and their average age was 74 years. They could perform lower limb exercises, including ankle movements during the bed rest period. Twenty-three patients did not complain of pain during LEX exercise. Vertebral fractures are more common in the elderly, such as the patients in this study, and they are at a high risk of thromboembolism [5,16-18]. Nonetheless, no symptomatic thrombi were observed in this study. Moreover, some patients with vertebral fractures requiring bedfast for conservative therapies hoped to perform LEX exercises for a longer time and longer period. We hope that they enjoyed the exercises and realized the importance of VTE prevention. In addition, the recommended treatment for vertebral fractures varies according to guidelines, and the optimal method is not clear [19]. Surgical treatment allows early ambulation, and voluntary lower extremity exercises with LEX may be unnecessary. However, there is a concern that the bed rest period may be prolonged with a longer surgical waiting period, when patients cannot be released immediately after surgery or when conservative therapy is used. In such cases, the LEX may motivate patients to perform voluntary lower extremity exercises, which may be useful for thromboprophylaxis. Based on these facts, it seems useful to introduce the LEX exercise program to patients with vertebral fractures to encourage them to move their legs during the bed rest period.

This study protocol was considered feasible and safe. The risk of VTE in elective spinal surgery patients ranges from 0% to 31% [4]. Neither severe adverse events nor symptomatic VTE occurred in this study, and over 90% of the patients completed the protocol. Average D-dimer levels around the end of the LEX program were much lower than those prior to completing the LEX program. The decrease in D-dimer levels may be due to a period post-injury, but it could also result from exercise using the LEX. Diastolic blood pressure was significantly different before and after the first exercise session. There was also a significant difference in the modified Borg scale scores for breath and legs before and after the first exercise session and for legs before and after the last exercise session. However, no clinically significant changes in vital signs were observed before and after exercise in both first and last sessions. The criteria for discontinuation of rehabilitation by the Japanese Association of Rehabilitation Medicine include tachycardia of ≥140 bpm and increases in systolic and diastolic blood pressure of 40 and 20 mmHg or more, respectively, during exercise [20]. In this study, no patients reached these discontinuation criteria during the first or last LEX session.

Both mechanical and pharmacological methods are recommended to prevent VTE [8]. However, it is difficult to perform pharmacological thromboprophylaxis in patients with bleeding risk, such as those undergoing spinal surgery. Therefore, the LEX may be useful with regards to strengthening mechanical thromboprophylaxis [5].

Cancer patients and pregnant women are at a high risk of VTE [21,22]. Therefore, they may be less active. We would like to use the LEX for patients with other diseases, such as cancer or pregnant women, and evaluate its usefulness.

Finally, the world is currently facing a pandemic. Coronavirus disease 2019 (COVID-19) has become a pandemic and increased the risk of VTE [23]. Moreover, COVID-19 is an infectious disease that requires isolation, reducing the chances of getting out of bed and rehabilitation and increasing the risk of VTE. Patients can exercise using the LEX by themselves, which may help prevent VTE and secure opportunities for exercise instead of rehabilitation.

There are some limitations to this study. First, it is difficult to prove the effectiveness of the LEX in preventing VTE because the sample size was too small. Second, the effectiveness of the LEX alone is unclear because, in this single-arm study, we used other thrombosis prevention devices, including GCS and IPCD. Third, we would like to study the effect of LEX exercise on maintaining muscle strength or activities of daily living improvement in the future. Finally, for the LEX to become more popular, some improvements are needed. For example, it should be made lighter and easier to be set up by patients themselves.

## Conclusions

This is the first study regarding mechanical thromboprophylaxis through in-bed exercise for patients with spinal disease who are required to be bedfast. Exercises using the LEX for bedfast patients due to spinal disease were well accepted and associated with no severe adverse events. Additionally, no symptomatic VTE occurred in these patients. The results of this study suggest that the protocol of exercise with the LEX to prevent symptomatic VTE is feasible and safe in patients on bed rest due to spinal disease. Therefore, we believe that exercises using the LEX can be useful for patients on bed rest due to various diseases. Moreover, not only medical experts but also patients could notice the importance of voluntary leg movement for thromboembolism prophylaxis through exercise using the LEX.

## Appendices

Appendix A

**Table 7:** The data of D-dimer level before starting the LEX protocol and after the LEX protocol Blank space means that no data was measured. LEX: Leg exercise apparatus

	Before starting the LEX protocol	After the LEX protocol
Subject	D-dimer	D-dimer
1	4.3	4.1
2	7.9	
3	5.6	1.8
4	1.6	1.8
5	3.8	3
6	6.4	0.9
7	1	0.9
8	1.2	1.6
9	8.1	4.9
10	27.2	0.8
11		2.3
12	1.8	
13	1.3	
14	5.4	1.3
15	3.1	3.6
16	6.6	
17	2.7	2.7
18	5.5	5.5
19	3.3	14.4
20	38.8	
21	12.4	
22	12.9	3.6
23	3.7	4.9
24	2.9	1.4
25	33.9	1.3
26	1	0.8
27	2.9	5.2
28	1.1	
29	1.1	5.8
30	1.6	1.2
31	5.2	

**Table 8:** The data of circulatory dynamics before and after exercise using the LEX in the first exercise session Blank space means that no data was measured. 'Before' column features data collected before the LEX exercise. 'After' column features data collected after the LEX exercise. sBP: Systolic blood pressure [mmHg; dBP: Diastolic blood pressure [mmHg]; PR: Pulse rate [bpm]; SpO2: Saturation of percutaneous oxygen [%]; LEX: Leg exercise apparatus

	Before	After	Before	After	Before	After	Before	After
Subject	sBP	sBP	dBP	dBP	PR	PR	SpO2	SpO2
1	108	111	60	68	105	112	96	95
2	112	103	72	72	89	90	97	96
3	136	120	78	82	64	64	97	97
4		112		63		65		97
5	105	106	70	69	72	65	99	96
6	118	103	80	75	78	75		95
7	139	133	84	92	78	87	98	99
8								
9	131	144	86	100	83	82	95	95
10	150	152	73	75	73	70	98	98
11								
12	120	128	67	74	85	89	96	96
13	93	103			52	60	97	98
14	144	157	80	86	87	83	96	97
15	105	110	77	66	86	79	98	98
16	128	131	64	66	86	89	98	99
17	147	151	77	88	80	89	98	98
18	136	145	97	98	99	98	97	96
19	151	156	95	96	81	79	99	99
20	141	136	78	94	77	71	96	95
21	137	139	90	89	78	82	97	97
22	146	127	89	92	70	69	94	96
23	156	163	77	79	102	107	95	95
24	130	124	69	79	58	60	95	95
25	142	140	96	90	91	93	98	98
26	139	138	89	88	80	83	98	98
27	116	130	76	76	75	68	97	
28	132	135	77	76	62	62	95	96
29	122	124	64	58	58	58	97	97
30	109	116	64	67	80	82	98	97
31	157	160	77	78	76	78	97	98

**Table 9:** The data of circulatory dynamics before and after exercise using the LEX in the last exercise session Blank space means that no data was measured. 'Before' column features data collected before the LEX exercise. 'After' column features data collected after the LEX exercise. sBP: Systolic blood pressure [mmHg; dBP: Diastolic blood pressure [mmHg]; PR: Pulse rate [bpm]; SpO2: Saturation of percutaneous oxygen [%]; LEX: Leg exercise apparatus

	Before	After	Before	After	Before	After	Before	After
Subject	sBP	sBP	dBP	dBP	PR	PR	SpO2	SpO2
1								
2								
3								
4								
5								
6								
7								
8								
9	126	128	72	76	72	74	98	98
10								
11								
12	108	111	62	66	89	88	98	98
13								
14	118	120	64	66	73	74	97	97
15	126	126	64	65	68	70	98	98
16	120	121	59	55	90	92	98	98
17	112	121	72	70	78	80	98	98
18	132	134	78	78	96	98	98	98
19	129	130	85	80	81	80	99	99
20	125	135	72	81	70	91	96	97
21	121	124	78	76	75	77	98	98
22	120	118	62	52	63	62	97	97
23	164	169	67	70	72	66	98	97
24	118	112	76	77	56	56	97	97
25	148	142	79	78	82	80	98	98
26	148	142	82	76	82	88	98	98
27								
28								
29	116	118	62	64	56	58	98	98
30	114	117	62	68	75	78	98	98
31	114	120	63	70	66	68	98	98

**Table 10:** The data of circumference of lower limb on the first day and the last day of the LEX exercise Blank space means that no data was measured. 'First' column features data from the first session of the LEX exercise. 'Last' column features data from the last session of the LEX exercise. The maximum calf circumference was taken in cm. The thigh circumference (cm) was measured 10 cm above the patella.

	First	Last	First	Last	First	Last	First	Last
Subject	Right calf	Right calf	Right thigh	Right thigh	Left calf	Left calf	Left thigh	Left thigh
1	25	24.5	28	31.5	25	24.5	30	29.5
2	30.5	29.5	41	40	30	30.5	41.5	40.5
3	28.5	28.5	38	37	28.5	29	36	37
4	31.3		37.5		30.8		37	
5	28		35.5		27.5		35.5	
6	28.5		36.5		28		37	
7	23.5		31		23.5		31	
8	29.4	28.5	41.2	37.7	30.5	29.5	41	37.3
9	32.5	33	45	45	33	33	45	45
10	25.8		33		26.1		32.7	
11								
12	25	24.5	29.5	29	25	24.5	28.5	29
13	27.5		32.5		28		33	
14	31	31	40	40.5	31.5	31	40	40.5
15	35.5	35.8	43.5	43.5	36.5	35.5	44.5	45
16	29.5	28.5	35	34	29	28	35	35
17	23.5	23	28.5	28	23.5	23	29.5	29
18	28	27.5	42.5	43	28	28	38	38.5
19	29.4	38.5	34.8	35	29.5	38.7	35.7	35.2
20	32	31.1	38.9	38.3	32.5	32.1	40	36.9
21	31	31.2	39	38.8	31	31	38.5	38.5
22	32.6	32.5	40.3	39.8	32.1	32	40.3	39.5
23	31	28.7	34	34.5	31	29.5	35	34.5
24	36.9	35.1	43.7	43.2	36.8	33.1	44	44.3
25	32	31.5	39.5	39.3	32	31.3	39	39.2
26	27.5	27.5	36	36	27.5	27.5	36	36
27	28.5		32.5		29.5		34.5	
28	31.5		36		31		36	
29	37.5	37.5	49	48.5	38	37.5	49	48
30	35.5	35	43.5	43.5	35	35	43.5	43
31	35.5	33.5	41.5	40	36	33	41.5	40
